# Attitudes of Mental Health Professionals Towards the Use of Routine Outcome Monitoring in Psychotherapeutic Inpatient Settings: A Thematic Analysis

**DOI:** 10.1007/s10488-025-01455-w

**Published:** 2025-07-01

**Authors:** Julia Barbara Krakowczyk, Martin Teufel, Eva-Maria Skoda, Christoph Jansen, Tania Lalgi, Lennart Martens, Ulrike Dinger, Wolfgang Lutz, Alexander Bäuerle

**Affiliations:** 1 Clinic for Psychosomatic Medicine and Psychotherapy, LVR-University Hospital Essen, University of Duisburg-Essen, Essen, Germany; 2 Center for Translational Neuro- and Behavioral Sciences (C-TNBS), University of Duisburg-Essen, Essen, Germany; 3 Department for Psychosomatic Medicine and Psychotherapy, Medical Faculty, LVR Hospital Düsseldorf, Heinrich Heine University Düsseldorf, Düsseldorf, Germany; 4 Clinical Institute for Psychosomatic Medicine and Psychotherapy, Medical Faculty and University Hospital Düsseldorf, Heinrich Heine University Düsseldorf, Düsseldorf, Germany; 5 Department of Clinical Psychology and Psychotherapy, University of Trier, Trier, Germany

**Keywords:** Outcome Measures, Precision Mental Health Care, Precision Psychotherapy, Feedback-Informed Psychotherapy, Personalized Psychotherapy

## Abstract

Routine outcome monitoring (ROM) involves the systematic recording of patient-reported outcome measures to monitor treatment progress and outcomes within psychotherapy. It represents an evidence-based approach to enhance psychotherapeutic treatment outcomes. However, it remains a rather controversial topic among mental health care professionals (MHP), resulting in its limited use in clinical practice, especially in inpatient settings. This qualitative interview study aimed to gain a comprehensive understanding of MHP attitudes towards ROM and its implementation within psychotherapeutic inpatient settings in Germany. In this study, semi-structured interviews were conducted with MHP working in the psychotherapeutic inpatient setting in the German healthcare system. Three independent researchers inductively coded the data, and thematic codebook analysis was used to evaluate the data iteratively. In total, 20 participants (14 medical doctors, and 6 clinical psychologists) with varying levels of working experience participated in the present study. The general attitude towards ROM and its implementation was predominantly positive, with ROM being perceived as a helpful feature to support psychotherapeutic inpatient treatment. However, possible pitfalls requiring careful consideration were highlighted, such as the risk of potential misuse and additional workload. The insights of the present study contribute to the ongoing discourse of ROM in mental health care. The study highlights the importance of proper implementation strategies and transparent communication regarding the relevance, aims, and use of ROM. Moreover, it highlights potential risks and perceived disadvantages associated with ROM, which may be related to implementation barriers and possible negative attitudes among MHP.

**Supplementary Information:**

The online version contains supplementary material available at 10.1007/s10488-025-01455-w.

## Introduction

Routine Outcome Monitoring (ROM) has gained prominence in mental health care (Carlier & Eeden, 2017; de Jong et al., 2012; Delgadillo et al., 2018). It involves the systematic and repeated assessment of patient-reported outcome measures throughout treatment, allowing mental health care professionals (MHP) to monitor progress, tailor interventions, and optimize patient care (Lambert & Harmon, 2018; Lambert et al., 2018; McAleavey et al., 2024; Muir et al., 2019). This feedback-based system allows MHP to track treatment progress and adapt interventions, fostering personalized and precision psychotherapy (Dobud et al., 2020; Tilden & Whittaker, 2022). It has been shown that ROM can enhance the effectiveness of psychotherapy through MHP receiving feedback on the progress or potential failure of psychotherapeutic treatment (Lambert et al., 2018). Further research has shown that patients had a 2.5 times higher likelihood of showing psychotherapeutic treatment progress while assigned to the ROM condition compared to patients in the treatment-as-usual (TAU) condition (Brattland, 2018). Recognizing the deterioration or non-responsiveness in the therapeutic progress has been shown to significantly reduce deterioration and dropout rates during psychotherapeutic treatment (Anker et al., 2009; Kraus et al., 2011; Lambert et al., 2018).

Within psychotherapy research, a paradigm shift towards more patient-centered care is evolving, in which the integration of ROM into clinical practice becomes increasingly significant (Lutz et al., 2015). This paradigm shift, however, is not as present in clinical practice, and only a small minority of MHP (13%) are required by their institutions to collect standardized outcome measures for all patients routinely (Wiebe et al., 2021). Surveys in different countries have shown that routine outcome monitoring is not used by many mental health care professionals (MHP) despite its advantages (Puschner et al., 2015; Boswell et al., 2015; Winkeljohn et al., 2017; Campbell et al., 2023; Jensen-Doss et al., 2018). In general, there are only a few countries, such as the Netherlands (van Sonsbeek et al., 2021), Australia (Oster et al., 2023), Canada (Jonášová et al., 2024), and New Zealand (Jonášová et al., 2024; Smith & Baxendine, 2015), that have routinely incorporated ROM into their mental health care system. Upon closer examination of the situation in Germany, despite few testing trials, there is no nationwide or trans-sectoral implementation of ROM in the mental healthcare system (Puschner et al., 2015; Ross et al., 2016).

ROM becomes particularly relevant in psychotherapeutic inpatient settings (i.e. hospitalized treatment in mental health hospitals), where the intensity and complexity of care often necessitate vigilant assessment and adjustment (Lewis et al., 2019). Overall, psychotherapeutic treatment in inpatient settings has been critical for decades and can hardly be intercepted by individual institutions in Germany (Kowitz et al., 2014; Rommel et al., 2017). Also, patients being treated in inpatient units require more intense treatment due to a large symptom burden or severe crisis (Goldstein & Horgan, 1998; Loch, 2014). Therefore, treatment resources are scarce and demands are high (Plötner et al., 2022) and the implementation of ROM could hold the potential to not only improve treatment outcomes but also contribute to more efficient resource allocation within psychotherapeutic inpatient treatment (Boswell et al., 2015; McAleavey et al., 2024; Muir et al., 2019; McAleavey & Moltu, 2021; Azizian et al., 2024).

While the efficacy of ROM is well documented in research, understanding the attitudes of MHP towards this approach in clinical practice is equally crucial. This is particularly important, as MHP acceptance is related to the actual use of evidence-based approaches (such as ROM) in clinical practice (Hildebrand et al., 2023; Planert et al., 2025; Rye et al., 2019). Overall, the existing research regarding MHP attitudes towards ROM and its implementation is somewhat contradictory. While some research found positive attitudes towards its use and implementation (Jensen-Doss et al., 2018; Valdiviezo-Oña et al., 2024), other research reported large criticism and even rejection among MHP (Buwalda et al., 2015; de Beurs et al., 2011; Kaiser et al., 2018). Moreover, the process of implementing and regularly using ROM can be challenging and is associated with ambivalent attitudes among MHP (Gelkopf et al., 2021; Van Wert et al., 2020). Overall, existing research suggests that the use of ROM in clinical practice displays a rather sensitive and controversial topic among MHP.

It is important to note that research investigating attitudes towards ROM has been primarily conducted in psychotherapeutic outpatient settings (Gelkopf et al., 2021). Hence, there is little research focusing on attitudes towards ROM in the inpatient setting (Van Wert et al., 2020; Lambert et al., 2003, 2018). This might be related to organizational difficulties and barriers of implementing ROM in inpatient settings, which is why it is not commonly used in inpatient settings (Lampis & Rocca, 2024; Van Wert et al., 2020; Trauer et al., 2009). Particularly in Germany, there is a large research gap investigating ROM in inpatient settings and MHP’s attitudes towards it (Puschner et al., 2015). As the inpatient treatment is very distinct from outpatient treatment (Goldstein & Horgan, 1998), the existing research on MHP attitudes towards ROM cannot be generalized across settings. Also, patients being treated in inpatient settings experience higher symptom burden and more complex psychopathology (Goldstein & Horgan, 1998; Doering et al., 2023; Loch, 2014), so the requirements for a ROM system differ. As a result, the adaptation of ROM and specific barriers in implementation differ, which can possibly affect the attitudes of MHP towards this approach as well.

The present study aims to explore attitudes towards ROM and its implementation of MHP working in psychotherapeutic inpatient settings. The specific goals are to gather a general sentiment towards this approach and to identify perceived benefits as well as challenges and barriers associated with ROM and its implementation. Taking on a qualitative approach, the present study aims to explore attitudinal domains as well as specific needs and preferences of MHP regarding ROM in depth. By gaining a nuanced understanding beyond the measures of quantitative data, the results gathered from the present study not only contribute to the field of implementation research but also can further support the development of health policy recommendations for ROM in mental health care.

## Methods

### Participants and Researchers

The participants consisted of MHP working in inpatient psychotherapeutic units. All MHP had different working experience, ranging from under one year to more than five years. The MHP had different psychotherapeutic orientations (cognitive-behavioral therapy, psychodynamic psychotherapy, and systemic psychotherapy). Experience with ROM was not a pre-selected requirement for study inclusion. Inclusion criteria consisted of 1) working as a MHP in a psychotherapeutic inpatient unit and 2) conducting psychotherapy.

Purposive sampling was carried out. In total, 23 MHP were contacted by email, of whom 20 agreed to participate in the study and 3 did not respond. Data analysis started during participant recruitment and stopped after reaching thematic saturation (Guest et al., 2016). Based on Charmaz (2006), saturation was achieved when no new properties emerged from the obtained data. MHP working in different university medical centers were contacted to achieve a diverse range of clinical experience and working preferences. The sample consisted of medical doctors, and clinical psychologists, as these occupations conduct psychotherapy in inpatient units in Germany.

The researchers in this qualitative study had a research background in clinical psychology, psychosomatic medicine, and psychotherapy with different research experiences (full-degree professors, post-doctoral researchers, Ph.D. candidates, and research assistant). All researchers had a rather positive but critical attitude towards ROM in the inpatient setting.

### Study Design

The present study comprised a cross-sectional qualitative research design with semi‐structured interviews of MHP and the evaluation of their attitudes towards ROM and its implementation. Ethical approval has been obtained by the ethical committee of a medical faculty. Data collection took place between 11/2023 and 01/2024. Data was collected from 20 MHP. This study was conducted in line with the Consolidated Criteria for Reporting Qualitative Research (COREQ) checklist (Tong et al., 2007) and followed accepted guidelines to ensure quality and validity in qualitative research (Yardley, 2000). The COREQ guideline can be found in Appendix A. The participants were recruited from two university medical centers specializing in psychosomatic medicine and psychotherapy. These medical centers were selected because they provide complex psychotherapeutic inpatient treatment in Germany. A standardized ROM system has not been implemented yet. However, both medical centers were working on an implementation strategy.

### Semi-Structured Interview

A semi-structured interview was developed based on the Evidence-based Practice Attitude Scale (EBPAS)-ROM by Rye et al. (2019) as well as self-generated questions. The interview first underwent a pilot test to assess the clarity of the formulated questions and to estimate the approximate duration of the interview. Throughout this iterative process, questions were refined and modified as necessary. The EBPAS-ROM questionnaire is a validated instrument that measures provider attitudes towards ROM, providing a solid foundation for the interview questions. An interview format was chosen, as the aim of the study was to gather in-depth information regarding MHP attitudes towards ROM beyond the boundaries of quantitative data. The research team was interested in hesitations and specific reasons behind negative attitudes as well as inter- and intrapersonal nuances, which is why an interview format was perceived as suitable. Moreover, this format provided room for discussion and allowed MHP to provide information that they believed was important and relevant (Brennan & Stevens, 1998).

The interview began with sociodemographic questions (e.g. age, gender, employment duration, clinical experience, and primary responsibilities within the institution) and proceeded to explore participants'attitudes and experiences with ROM. In the beginning, participants were asked to define ROM and discuss their past encounters with it. In both cases, whether participants expressed unfamiliarity or familiarity with ROM, a standardized definition of ROM, based on Lambert et al. (2018), was offered to ensure consistency in subsequent discussions. Fifteen self-generated questions based on the EPBAS-ROM Scale were tailored to the research objectives, covering topics such as the purpose of using standardized assessment instruments, previous use of assessment instruments in inpatient settings, attitude towards the ROM approach and its implementation, and prerequisites for possible implementation. Open-ended questions and prompts were used to facilitate discussion, ensuring comprehensive data collection. The interview questions and the provided definition of ROM are provided in the supplementary material (S2).

### Data Collection

Both verbal and written descriptions of the study were provided and written informed consent was obtained. Once enrolled in the study, each participant took part in a 30-min semi-structured interview. Twelve interviews were conducted digitally and eight face-to-face. During the interview, there was no presence of non-participants. The interviewer took field notes. Data collection stopped upon achieving theoretical saturation, indicating that no new properties emerged from the obtained data (Charmaz, 2006). In the present study, saturation was reached with a total of 20 interviews to develop a richly textured understanding of the attitudes towards ROM in the psychotherapeutic inpatient setting (Hennink et al., 2016). No modification of procedures was required as part of the data collection. The auditory data was stored on a password-protected terminal device and saved for the next 10 years. No prior relationship has been established between the interviewer and the participants prior to the study. No characteristics about the interviewer were reported to the participants.

### Quality Control

The COREQ checklist, presented in the supplementary material (S1), has been followed. Based on Kuckartz (2018), reliability was established through independent coding of the data by two independent researchers and a final cross-coding procedure by another independent researcher. The lack of concordance was resolved through discussion with the researchers throughout the iterative process. For the iterative process, a reflexive journal was maintained, and the researchers met regularly to discuss the research and analysis. A thorough and detailed account of the location, the participants, and the limitation of a qualitative study is delivered to build credibility (Hussy, Schreier, & Echterhoff, 2013). Guidelines for constructivist research were followed through maintaining a reflexive journal, extensive engagement with the data, as well as dialogue between the research team to ensure the validity of this current study (Creswell & Miller, 2000). Peer debriefing was used as a means of requesting objective support throughout validation procedures.

### Data Analysis

The dataset comprised 20 pseudonymized transcripts. The duration of the interviews ranged from 18 to 36 min (*M* = 26 min, *SD* = 4 min). Transcriptions were performed by f4x (Audiotranskription, 2024), ensuring accuracy, and subsequently reviewed for data confidentiality following ethical guidelines. After transcription, identifiable information was removed, and audiotaped data was securely stored. The MAXQDA software (VERBI, 2021, version 2024) has been used for facilitating systematic organization and analysis. Thematic analysis, following Braun and Clarke's (2006) six-step iterative process, was conducted at a semantic level in MAXQDA to identify patterns related to the research aim. At the start of the process, the transcripts had to be read repeatedly and initial ideas noted down. In a second step, initial codes were then systematically created for the entire data set. These codes were then organized in the third step by examining patterns and relationships between the codes to identify possible themes. In the fourth step, these themes were reviewed to ensure that they accurately reflected the coded data and the dataset as a whole. In the fifth step, the themes were defined and clearly named to capture their essence and relevance to the research questions. Finally, the themes and subthemes were further refined and finalized in order to reflect their overall significance and to ensure that they provide a consistent representation of the results.

Two independent researchers, ensuring coding consistency, conducted the data analysis independently. Afterwards, another third researcher independently cross-coded the data. Afterwards, the final themes were reviewed and verified by the research team. Thematic analysis, following Braun and Clarke's (2006) six-step iterative process, was conducted at a semantic level in MAXQDA to identify patterns related to the research aim.

A constructivist paradigm was adopted in this study, reflecting the belief that knowledge and reality are co-constructed through the interactions between the researcher and participants. By applying this paradigm, the subjective meanings, and interpretations that individuals attach to their experiences were emphasized, which aligns with the study's aim to understand participants'attitudes and perceptions towards ROM (Creswell & Poth, 2018). The inductive approach by analyzing bottom-up allowed themes to emerge directly from the data rather than fitting the conducted data into pre-existing categories (Braun & Clarke, 2006). This approach enabled to capture the nuanced and diverse perspectives of the participants, allowing for a richer understanding of ROM in the psychotherapeutic inpatient setting.

## Results

### Subjects

The ages of participants ranged from 25 years old to 57 years old (*M* = *35* years, *SD* = 8 years), of which two identified as a man and 18 as a woman. Sociodemographic characteristics of the sample can be found in Table 1.

**Table 1:** Sociodemographic Characteristics of Participants

Characteristic	Values	
	N	(%)
Gender		
Identified as female	18	(90)
Identified as male	2	(10)
Age, in years		
20–29	7	(35)
30–39	7	(35)
40–49	5	(25)
50–59	1	(5)
* Mean*	*35.05*	
* SD*	*8,92*	
Working experience in inpatient setting		
< 1 year	6	(30)
1–3 years	4	(20)
3–5 years	4	(20)
> 5 years	6	(30)
Psychotherapeutic specialization		
Cognitive-behavioral therapy	10	(50)
Psychodynamic psychotherapy	9	(45)
Systemic psychotherapy	1	(5)
Executive function		
Yes	9	(45)
No	11	(55)
Knowledge regarding ROM		
Yes	12	(60)
No	5	(25)
Not sure	3	(15)
Occupation		
Medical Doctor	14	(70)
Clinical Psychologist	6	(30)

*N* = 20

### Emerging Themes

**Fig. 1:**
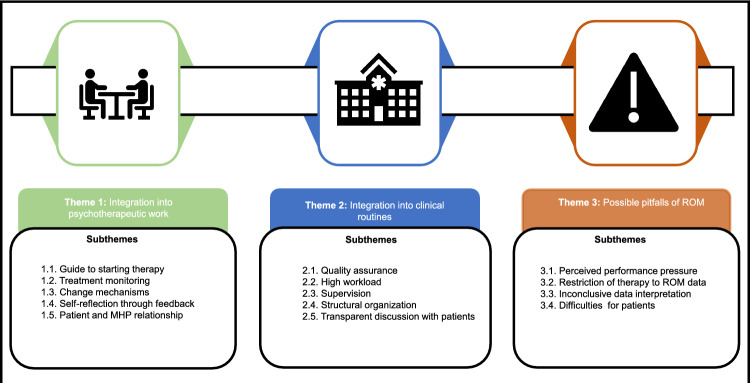
Themes and subthemes from the codebook analysis. Graphic display of the overarching themes, and subthemes that emerged during the iterative data analysis process

Three major themes emerged from the data: (1) integration into psychotherapeutic work with five subthemes, (2) integration into clinical routines with five subthemes, and (3) possible pitfalls of ROM with four subthemes. A graphical representation of the emerged themes and subthemes can be found in Fig. 1.

## Theme 1: Integration into Psychotherapeutic Work

This theme explores participants'views on the importance of using standardized assessment instruments during inpatient treatment. Ninety percent of the participants (18 of 20) stressed the importance of integrating these assessments into clinical practice, thereby highlighting the psychotherapeutic value of ROM.

### Subtheme 1.1: Guide for Psychotherapy

*“(ROMs) are important and perhaps bring a little more focus to the therapy. Keyword red thread.”*

ROM was perceived as helpful for treatment planning and direction of the treatment, as it offers further information about the patient and their symptom burden (see quote [1]). In this regard, ROM was perceived as a valuable feature for bringing additional structure and focus to the psychotherapeutic process. One participant noted that ROM could help provide a clearer framework regarding the course of therapy, thereby giving it more direction (see quote [2]). Another participant highlighted that ROM could bring a"red thread"or consistent focus to therapy, ensuring that both therapist and patient remain aligned on treatment goals (see quote [3].). It was also discussed that ROM would shift the regular psychotherapeutic inpatient treatment to a more “personalized medicine” approach (see quote [4]). Moreover, it was mentioned that ROM could be especially useful for MHP with less working experience, as it can offer further guidance and structure (see quote [5;6]). This subtheme reflects the potential of ROM to support structure and clarity within the process of psychotherapy.

### Subtheme 1.2: Treatment Monitoring

*"You also look at the lab values relatively often or you monitor any other values like an ECG or whatever. And why shouldn't we do that on a psychometric, diagnostic level?"*

One of the key perceived advantages of integrating ROM in the inpatient setting, mentioned by 60% (12 of 20), was the possibility having access to a detailed monitoring of patients regarding their symptoms and level of functioning next to the clinical evaluation. In this regard, ROM was identified as a useful feature for treatment monitoring. One participant compared ROM as a possibility for standardized objective monitoring and assessment within the field of psychosomatic medicine and psychotherapy, thereby drawing an analogy to EEG or blood tests (see quote [7]). Also, this monitoring was described as useful as it was perceived to generate a feeling of being more “in control” regarding the course of treatment (see quote [8]). This subtheme reflects ROM as a measurable structure for diagnostics and treatment monitoring within psychotherapeutic inpatient treatment.

### Subtheme 1.3: Change Mechanisms

*“The greatest added value is simply that we get an insight into something that was previously a bit of a black box.”*

Participants highlighted the value of ROM in providing greater transparency and insight into the psychotherapeutic process by „being able to identify mechanisms of action” (1. interviewee) and “making mental stress visible” (5. interviewee). One participant emphasized that ROM could help to uncover aspects of psychotherapy that were once considered a"black box,"offering clearer visibility regarding patient progress and treatment effectiveness (see quote [9]). The insights gained from ROM would also enable patients to get an overview of what happens in psychotherapy and if measurable changes occurred (see quote [10]). Multiple participants highlighted the use of ROM as a possibility to generate new knowledge relevant to both science and clinical practice. This subtheme highlights the possibility to use ROM as an approach to detect dynamics within psychotherapy such as the course of recovery during inpatient treatment.

### Subtheme 1.4: Self-Reflection Through Feedback

*"You often have a distorted perception of how the therapy process is going and sometimes the therapist has the impression that it's going much better than the patient would think."*

ROM was compared to"mini-interventions"(1. interviewee), stressing that its active use in therapy could draw patients'attention to issues they might not have previously considered, thereby fostering introspection (see quote [11]). Furthermore, participants recognized ROM as a valuable tool for enhancing their self-reflection in clinical practice. One participant noted that the use of assessment instruments increased their vigilance in patient interactions, making them more aware of subtle dynamics (see quote [12]). Another pointed out that ROM can help to identify and address potential discrepancies between the MHP and the patient's perceptions of the treatment progress (see quote [13]). This subtheme highlights the potential of ROM to foster MHP self-reflection through feedback.

### Subtheme 1.5: Patient and Therapist Relationship

*“At the moment I see it more as a feature that can perhaps be quite positive for my therapeutic development and also for the relationship with the patient”*

Sixty-five percent of the participants (13 of 20) stressed the importance of the therapeutic alliance as one of the major components of the therapeutic process. The opinion regarding ROM and its influence on the therapeutic alliance was contradictory. On the one hand, some participants voiced concerns that discussions related to ROM might reduce the time available for therapeutic interactions, potentially affecting the depth and quality of patient engagement. On the other hand, others suggested that ROM could positively contribute to their therapeutic development and improve their connection with patients (see quote [14]). In this regard, the belief that ROM strengthens the therapeutic relationship by encouraging deeper reflection on this crucial aspect of therapy was expressed (see quote [15]). This subtheme highlights the importance of properly implementing ROM according to the MHP and patient needs to not disrupt the therapeutic alliance but rather implement it in a way so that it can foster the therapeutic alliance.

## Theme 2: Integration into Clinical Routines

This theme highlights the perceptions about the incorporation of ROM into the clinical routines of the inpatient setting. Overall, the implementation of ROM in the inpatient setting was seen as"a great opportunity."(5. interviewee). Out of the participants, 70% (14 of 20) believed that the implementation of ROM would lead to significant benefits. They felt that ROM would offer valuable insights into patient progress and outcomes, thereby supporting its integration into the clinical routine.

### Subtheme 2.1: Quality Assurance

*"And we no longer have to rely solely on this therapeutic sovereignty, where we have a feeling and then make ourselves like this, but that we have measured this objectively. So I think objectivity really is the big opportunity."*

Eighteen out of 20 (90%) participants described objective measurement as the most important advantage of ROM. ROM assessments were considered as an objective measure that could provide valuable data and insights. In this regard, ROM was considered as a “means for quality assurance” (2. interviewee) for inpatient treatment (see quote 16; 17]). It was highlighted that data generated from ROM could guide and improve therapeutic decision-making, thereby enhancing the quality of the treatment. This subtheme highlights the potential of ROM to serve as a means for quality assurance within the inpatient setting.

### Subtheme 2.2: High Workload

*“And I already know that there's a lot to do in everyday life on the ward and that's just on top of everything else, so to speak.”*

15 out of 20 participants (75%), expressed concerns regarding the scarcity of time due to an already high workload. The difficulty of actually using ROM data given their busy work schedules has been discussed. In this regard, concerns were voiced regarding ROM being possibly associated with “additional work” (see quote [18; 19]). This was described as a possible obstacle to actual usage (see quote [20]). The importance of selecting ROM measures with appropriate length and frequency was highlighted (see quote [21]). Overall, the perceived time constraints in a working environment with a high workload were identified as a major obstacle that needed to be considered.

The limited time available during therapy sessions to discuss the ROM evaluations with patients was also highlighted. Overall, 15 out of 20 participants (75%) voiced concerns that ROM could be particularly time-consuming, further emphasizing the need for organizational support and resource allocation to successfully implement ROM. This subtheme reflects the gap between optimal clinical practices in contrast to real life due to time constraints. It highlights the importance of implementing ROM efficiently in order to not lead to an enhanced workload.

### Subtheme 2.3: Supervision

*"I would definitely need comprehensive training for employees, including myself, on how to interpret this."*

The importance of proper supervision and training for ROM and its interpretation was mentioned by 13 (65%) participants. Participants emphasized the need for proper training particularly in interpreting the results of ROM assessments (see quote [22]). ROM supervision was seen as crucial to ensure that the MHP can feel confident and competent in utilizing ROM as part of their work (see quote [23]). Six participants (30%) would find regular ROM supervision sessions helpful in addition to ROM training. One participant suggested that incorporating ROM into their supervision would be beneficial, helping MHP to better understand and interpret the data (see quote [24]). This subtheme reflects the importance of embedding ROM in the context of MHP supervision, as the effectiveness of this approach is not only dependent on gathering data but also meaning making and interpretation.

### Subtheme 2.4: Structural Organization

*"I think it needs the structure where it routinely belongs. It needs structuring. Certain periods of time during the day, so that it's marked in the daily routine, plan, patients' timetable, what exactly time is set aside for it, because otherwise it goes down quickly."*

More than half of the participants (60%; 12 of 20) mentioned that the effective implementation of ROM is dependent on the presence of adequate infrastructure in the inpatient unit. In this regard, the importance of structural organization and suitable spatial conditions were highlighted. To achieve a structural organization, participants highlighted the need to integrate ROM as an integral component within the clinical routines. In this regard, twelve (60%) of the participants emphasized that the ROM assessment should be a fixed component within the patient's and MHP timetable, which is referred to in Table A3 as Quote (see quote [25; 26]). Routinely integrating ROM in the clinical workflow would help ensure that ROM is not overlooked and is handled as a regular part of the treatment.

Regarding suitable spatial conditions, a proper environment to fill in the ROM assessments was mentioned. It should be designed in a way that participants can fill in the assessments quietly without external disruptions, such as conversations among other patients, allowing them to engage thoughtfully (see quote [27]). In this regard, the lack of available space for patients to fill in the ROM assessment was described as a possible obstacle to ROM use (see quote [28]). Six participants (30%) voiced concerns about the availability of advanced and appropriate digital solutions for the effective implementation of ROM in their daily work routines. Additionally, participants highlighted the importance of accessible internet for patients to fill out questionnaires, highlighting the need for technological infrastructure. This subtheme underscores the importance of feasibility of a ROM system and the role of technological, organizational, temporal and spatial factors that are related to its implementation success.

### Subtheme 2.5: Transparent Discussion with Patients

*“I think it's always very important to discuss this with the patient, because they've completed the questionnaire and naturally want to know how they're getting on, so to speak, and discussing this often has a therapeutic purpose.”*

The importance of including patients in the aim of ROM and discussing the assessments regularly during their inpatient treatment has been highlighted. In this regard, transparency was highlighted as particularly important (see quote [29]). Transparency was described as important, as it might create uncertainty and unease among patients if they did not receive proper debriefing about the use of ROM (see quote [30]). Fourteen of 20 participants (70%) emphasized worries that patients might not complete ROM assessments consistently and reliably. They identified factors such as a lack of motivation and perceived lack of commitment as potential reasons for inconsistent data collection, which could challenge the comprehensive and precise use of ROM. Therefore, a mandatory debriefing to define ROM as an integral part of the inpatient treatment was mentioned as crucial. This subtheme highlights the importance of a dialogue between MHP and patients in regard to ROM and its interpretation.

## Theme 3: Possible Pitfalls of ROM

Although the overall attitude towards the implementation of ROM was very positive and it has been described as an approach with many advantages, clear concerns and risks associated with wrong implementation were highlighted.

### Subtheme 3.1: Perceived Performance Pressure

*“Well, for example, that therapists actually feel controlled, that patients feel controlled, what you do with these results. I mean, we have now, we are now here at the hospital, but there are also, I'll put it this way, private, commercially oriented providers, you could ask yourself whether there are consequences in terms of personnel planning and so on. So it's like a performance evaluation. Maybe you can say misinterpretation as a performance evaluation of the employees”*

Forty-five percent of the participants (9 of 20) expressed concerns that the implementation of ROM might create a sense of performance pressure among employees (see quote [31–33]). In this regard, participants voiced concerns about being judged based on ROM outcomes, fearing that consistently poor results could reflect negatively on their “work success” (see quote [34]). Also, the fear of receiving negative personal consequences (e.g. being fired) if the ROM data is being misused has been voiced. Also, participants mentioned that employees could feel controlled by ROM assessments. Overall, the perceived performance pressure and fear of being controlled associated with ROM implementation was expressed as a major concern. The importance of proper usage and focus on prevention of potential misuse has been highlighted. This subtheme reflects how a poor implementation of ROM may evoke pressure and anxieties among employees and the risk of ROM abuse as a surveillance tool.

### Subtheme 3.2: Restriction of Therapy to ROM Data

*“There is a concern that your own success will be measured by this, then of course you may start to only work with the patient when depression is queried, even though they might actually have two or three issues that would be more urgent, but are more individual and are not queried.”*

A concern raised by 20% (4 of 20) of the participants was that an over-reliance on ROM could restrict therapy by focusing predominantly on the aspects being measured, potentially overlooking other critical issues. Participants feared that when a measurement of “therapy success” is tied too closely to ROM, MHP might prioritize areas at the expense of other equally important, but unmeasured, patient needs (see quote [35]). The risks that ROM could narrow the therapeutic scope, reducing flexibility in addressing more individualized and urgent concerns have been mentioned (see quote [36]). This subtheme reflects the concern of ROM narrowing the psychotherapeutic focus. It further highlights the need of individualized therapy adapted to the patients’ needs and not to purely rely on ROM assessment.

### Subtheme 3.3: Inconclusive Data Interpretation

*“I'm actually worried that this will somehow lead to competition, but there may already be some comparability.”*

Participants voiced concerns regarding the interpretation of data obtained through ROM, particularly when it comes to drawing clear conclusions (see quote [37]). While some noted that ROM could help visualize progressions, making it easier to discuss therapeutic outcomes with patients, others were concerned that the data might lead to unintended consequences, such as fostering competition or encouraging comparisons among patients or MHP (see quote [38]). This subtheme highlights the risk of unintended social dynamics such as social desirability or competition with the implementation of ROM.

### Subtheme 3.4: Difficulties for Patients

*“If patients have such structural difficulties that simply performing regular ROMs is too much of a challenge.”*

A disadvantage concerning ROM covered the possibility that patients could not correctly use the ROM assessments. For example, one participant mentioned that patients might feel compelled to present themselves in a more favorable light in order to appear as though they are improving (see quote [39]). In this regard, ROM could be disadvantageous as patients who are highly focused on achieving improvement might feel disheartened or demotivated if ROM data suggests little or no progress, or even a decline in their condition, resulting in a mindset that their “own idea of performance cannot be satisfied” (1. interviewee). Another participant mentioned that ROM could also be used for particular criticism towards the treatment (see quote [40]). In addition, it was emphasized that the ROM might be seen by patients as a surveillance tool, which could negatively affect the patient-therapist relationship and create a potential obstacle to therapeutic progress (see quote [41]).

Four of the 20 participants (20%) expressed concerns about potential technical overload for patients when implementing ROM assessments digitally. One participant noted that patients with structural difficulties might struggle to consistently complete ROM assessments (see quote [42]). Another participant pointed out that the therapeutic relationship could be impacted by the nature of certain disorders, where patients may feel unseen or constrained, potentially affecting their engagement with ROM (see quote [43]). The concern is particularly relevant in cases where patients have complex or severe conditions, which could reflect negatively in their responses. Overall, 13 out of 20 participants (65%) identified disorder-specific impairments as a critical factor that should be considered when implementing ROM, especially with patients who are “structurally weak” (19. interviewee) or face significant cognitive or emotional challenges (see quote [44; 45]). This subtheme highlights the need of ROM to be specifically adapted to the patient population of the inpatient setting. It points out the relevance to adapt ROM to the level of functionality of the patient to avoid overload.

## Discussion

This study investigated MHP attitudes towards ROM and its implementation within the psychotherapeutic inpatient setting in Germany using thematic analysis. To the best of the authors’ knowledge, this is the first study that qualitatively investigated attitudes towards ROM of MHP working in the German psychotherapeutic inpatient setting. Three themes, namely (1) integration into psychotherapeutic work, (2) integration into clinical routines, and (3) possible pitfalls of ROM have been identified. The overall attitude regarding ROM was positive and it was perceived as a helpful approach to support psychotherapeutic inpatient treatment. While some participants anticipated significant generation of knowledge for science and clinical practice, others emphasized the supporting role of ROM in psychotherapeutic work. However, structural difficulties and possible pitfalls associated with implementation and usage were highlighted. In this regard, the importance of pre-defining the purpose and interpretation of ROM data and an efficient integration into the clinical workflow has been pointed out. The results of the present study contribute to both implementation science and clinical practice by offering a framework for an efficient adoption of ROM in mental health care from MHP perspectives.

In the present study, all MHP reported a predominantly positive attitude towards ROM and its implementation in the psychotherapeutic inpatient setting. This is partly in line with past research on MHP attitudes towards ROM, as the existing literature shows rather mixed results regarding attitudes of MHP regarding ROM and its implementation (Buwalda et al., 2015; de Beurs et al., 2011; Jensen-Doss et al., 2018; Kaiser et al., 2018). Particularly before standardized use, past research showed that attitudes among MHP tend to be rather negative which is in contrast to the results of the present study (Van Wert et al., 2020; Gelkopf et al., 2021).The contradictory research field highlights the sensitivity of this topic in clinical practice and suggests that even though ROM is considered an evidence-based approach, not everyone might perceive it as actually useful in clinical practice. This complexity is further underscored by contradictory opinions regarding ROM and its impact on the therapeutic alliance in the present study. It is therefore important to implement ROM as an additional feature, so that the MHP themselves are left to decide how to specifically include ROM into their work. Moreover, it highlights the importance of including MHP in the implementation process as their attitudes tend to be related to actual use.

The main benefits of integrating ROM comprised objectivity, an additional source of information, feedback system, structure, personalized treatment, and generation of knowledge. Particularly MHP with less working experience perceived ROM as a valuable feature, as it offered clear structure. This finding is in line with previous research showing more positive attitudes towards ROM usage among early career MHP (Hatfield & Ogles, 2004). Moreover, ROM was described as a helpful feedback system to detect discrepancies between MHP and patients’ perceptions regarding the treatment progress. This is in line with research showing indeed a discrepancy in perceptions between MHP and patients regarding psychotherapy-related aspects such as treatment progress or therapeutic alliance (Bachelor, 2013; Marziali, 1984). As these results indicate that the clinical judgement of MHP is not purely objective (Walfish et al., 2012), the implementation of ROM in the psychotherapeutic inpatient setting can offer a source of additional feedback from a patient’s perspective.

Considering the inclusion of the patient’s perspective into the psychotherapeutic inpatient treatment, ROM has been described as an opportunity to shift this type of treatment towards a more “personalized psychotherapy” or “personalized medicine” approach. This is in line with existing research supporting the ongoing paradigm shift towards personalized psychotherapy (also called “precision psychotherapy” or “enhanced psychotherapy”), in which the inclusion of data- and feedback systems plays a key role in psychotherapeutic treatment planning and actual treatment (Reese et al., 2024; Zipfel et al., 2024). Also, including the patient’s perspective in the inpatient treatment through ROM assessment has the potential to support shared decision-making (Joosten et al., 2011; Reese et al., 2024; Slade, 2017).

ROM was also perceived as a promising approach for quality assurance within the psychotherapeutic inpatient treatment in Germany. To date, there is no nationwide quality assurance system in psychotherapeutic inpatient units in Germany (Puschner et al., 2015). As the efficacy and cost-effectiveness of ROM have been proven, ROM has the potential to serve as a cost-effective feature for quality assurance (Barkham et al., 2023; Delgadillo et al., 2021; Lambert et al., 2018). This is in line with the recommendations of the American Psychological Association (APA) and the Joint Commission to include ROM as a means of quality assurance in mental health care (American Psychological Association, 2006; The Joint Commission, 2018). A standardized system for quality assurance becomes even more important in psychotherapeutic (partial-)inpatient treatment, as patients being treated in an inpatient setting in Germany display a high symptom burden and complex psychopathology, which is why a continuous monitoring is of high relevance to prevent negative outcomes (Doering et al., 2023).

In the present study, the disadvantages and possible pitfalls associated with ROM in the inpatient setting have been discussed. The most common concern mentioned included the feeling of being controlled through ROM. This was further described as the fear of ROM data being misinterpreted as a performance assessment for employees. Some MHP in the present study were concerned that their patients who are labeled as “not on track” could have negative personal consequences, such as being fired in the worst case. It is a very important concern that needs to be addressed when implementing ROM in clinical practice, as there is the risk that the ROM data can be misused as a performance assessment for MHP. The concerns mentioned in the present study are in line with existing research findings of similar worries among MHP, which was associated with a rather negative attitude during initial implementation processes (de Beurs et al., 2011; Kaiser et al., 2018). Moreover, it is in line with existing research highlighting the difficulties of adapting ROM due to attitudinal barriers among MHP (Barkham et al., 2023; Gelkopf et al., 2021; Rye et al., 2019). Taken together, it is of particular importance for leadership to be fully transparent about ROM and to ensure that the generated data will not be misused (i.e. the need of clear boundaries regarding data usage). Moreover, it highlights the important active involvement of MHP in the implementation process to foster trust and acceptance.

Another perceived pitfall of ROM was the concern that it might be too straining for some patients and not helpful for patients with more severe psychopathology, which is also in line with existing research stating that ROM might be associated with overload in patients with a lower level of functionality (Barkam et al., 2023; Rye et al., 2019). Also, the worry of an over-quantification of psychotherapy by focusing primarily on ROM data within treatment has been mentioned, which has been found in past research as well (Valdiviezo-Oña et al., 2024). In this regard, in line with existing research, the importance of adopting a more holistic definition of the terms recovery and treatment progress beyond the measures of quantitative data has been pointed out (Moltu et al., 2018). The worries and concerns related to ROM are important to consider, as the actual implementation process has been shown to display the greatest obstacle in ROM usage (Barkham et al., 2023). Thus, it is of high importance to pre-define the role of ROM in the inpatient treatment and to include the employees in the actual implementation process.

Practical implications that can be derived from the results of the present study include the general recommendation of the implementation of ROM in the psychotherapeutic inpatient setting. However, there is a need for clear transparency and communication regarding its use and a focus on the prevention of potential misuse. Moreover, ROM should be designed user-centered for both MHP and patients, so that ROM will be perceived as a helpful addition and not as a burden. Also, the specific integration of ROM into the psychotherapeutic work should be the MHP individual decision. As MHP working in inpatient units commonly experience a high working load and can experience exhaustion (O’Connor et al., 2018), ROM should be integrated as efficiently as possible, by e.g. integrating it into the hospital documentation system so that the assessment of the data is not associated with additional effort. Moreover, it is important to include the employees in the implementation process to foster acceptance. Governmental support (e.g. through funding) is important for the successful implementation of ROM in psychotherapeutic inpatient treatment, as many hospitals operate on limited budgets that hinder their ability to adopt such evidence-based practices (Dietrich & Riemer-Hommel, 2012). By providing financial resources, government support can help to facilitate the integration of ROM and enhance treatment outcomes.

## Limitations

The core limitation of the present study is that it investigates attitudes towards ROM prior to standardized use in psychotherapeutic inpatient settings. Even though previous research has shown that preliminary attitudes towards ROM tend to predict actual use (Rye et al., 2019), there is a possibility that attitudes change after actual implementation. Moreover, there is risk of sample bias, as the sample consisted of MHP working in university medical centers with a strong research focus. The strong research focus might have contributed to a generally positive attitude towards scientific approaches like ROM. Moreover, the working experience of the MHP has been assessed categorically, which does not allow for deeper exploration regarding working experience and attitudes towards ROM. Also, the limited length of the interviews (approximately 26 min) may have led to limited engagement and deeper exploration of this topic among MHP, potentially affecting saturation. Specifically, the study was conducted in two hospitals in an urban region, meaning that the results of the present study might not fully capture variability regarding the attitudes towards ROM among MHP working in different institutions and regions. Moreover, due to the cross-sectional design, inferences regarding the development and change of attitudes towards ROM over the course of implementation and usage cannot be made. As the field of implementation research of ROM is rather scarce in Germany, future research should focus on capturing a nationwide sentiment regarding ROM and its implementation among MHP using a standardized measurement instrument to assess attitudinal domains. This would allow for a deeper understanding of ROM and its acceptance as well as a possible adaptation to fit the MHP and patients’ preferences, thereby enhancing mental health care.

## Conclusions

The insights of the present study contribute to a nuanced understanding of MHP attitudes regarding ROM and its implementation of ROM in the psychotherapeutic inpatient setting. The qualitative approach facilitated an in-depth exploration of individual experiences and perspectives, contributing to a further understanding of the rather controversial nature of ROM in clinical practice. Overall, the attitudes towards ROM and its implementation in the present study were predominantly positive, with MHP identifying several advantages of integrating ROM in (1) the clinical workflow and (2) the psychotherapeutic work. However, the MHP also acknowledged possible pitfalls associated with ROM for both MHP and patients. It highlighted that it is crucial to perceive ROM as an additional feature in the inpatient treatment and not use it as a performance assessment. Moreover, the importance of tailoring ROM to the MHP and patients’ preferences and including MHP in the implementation process has been highlighted.

## Supplementary Information

Below is the link to the electronic supplementary material.

Supplementary file1 (DOCX 29 KB)

## Data Availability

Data that support the findings of this study are provided in the supplementary material. Further data are available from the corresponding author upon reasonable request.

## Declarations

### **Reporting guideline**

Consolidated criteria for reporting qualitative research (COREQ). The full COREQ-checklist is provided in the supplementary material.
