# Supplementary material for: Attitudes of Mental Health Professionals Towards the Use of Routine Outcome Monitoring in Psychotherapeutic Inpatient Settings: A Thematic Analysis
Source: Adm Policy Ment Health. 2025 Jul 1;52(4):771–84. doi: 10.1007/s10488-025-01455-w (PMC12310790; doi:10.1007/s10488-025-01455-w)
Supplement: Supplementary file 1 — Supplementary file1 (DOCX 29 KB) [file 10488_2025_1455_MOESM1_ESM.docx]

# **Supplementary Material**

**Table S1**

*Semi-structured interview.*

| Segments | Questions |
| --- | --- |
| Purpose of questionnaires in general | 1. What is the function of using questionnaires in the inpatient setting so far? 2. What purpose do these questionnaires have for you? |
| Use of questionnaires in the inpatient setting so far | 1. Who evaluated the questionnaire scores? 2. How were the scores presented to you? (e.g. raw scores, total scores, graphs etc.) 3. What role did the questionnaires play in the course of treatment? 4. Did you use the information obtained from the questionnaires only for yourself (as a therapist) or did you share them with your patients? |
| Acceptance of questionnaires in general | 1. Would you use questionnaires voluntarily?  - If yes, why- what are they helpful for? - If no, why not- what has been a hindrance so far? |
| General attitude towards ROM | 1. How do you feel about the implementation of ROM in our inpatient clinic?   **If negative attitude is present, further elaborate on this:**   - Why do you think it should not be integrated? - Could you elaborate on your reluctance towards ROM? - Is there anything you would be afraid of, if ROM would be integrated? - What would be needed to resolve your reluctance? |
| Specific questions ROM | 1. For which situations and/or patients do you think ROM can be of added value? What makes ROM concretely beneficial for your therapeutic work? 2. Can you imagine situations and/or patients where ROM use might form an obstacle? 3. Do you think using ROM would impact therapeutic proceedings? Would you like/dislike that and why? 4. Would you feel that that the use of ROM would change who you are as a therapist? |
| Conditions & Environmental Factors | 1. What conditions in our clinic do you think are needed for ROM to be actually integrated? 2. What environment would be required to fill in ROM questionnaires? (e.g. office, therapy room, quiet waiting area) 3. We think about using tablets or phones rather than paper-and-pencil, because of workload. What do you think? Would you have a different preference? Can you explain why? 4. Would you like to have trainings, webinars etc. for adapted use of questionnaires in your clinical practice? |

*Note.* Semi-structured interview that was conducted in the present study.

**Table S2**

*Consolidated criteria for reporting qualitative research (COREQ; Tong et al., 2007)*

| **Domain 1: Research team and reflexivity**  *Personal characteristics* | | **Reported on** |
| --- | --- | --- |
| 1. Facilitator | Julia Barbara Krakowczyk, Martin Teufel, Eva-Maria Skoda, Christoph Jansen, Tania Lalgi, Lennart Martens, Wolfgang Lutz, Ulrike Dinger, Alexander Bäuerle | Title page |
| 1. Credentials | Julia Barbara Krakowczyk (M.Sc.), Martin Teufel (Prof; MD), Eva-Maria Skoda (Prof; MD), Christoph Jansen (MD), Tania Lalgi (M.Sc.), Lennart Martens (B.Sc.), Wolfgang Lutz (Prof; PHD), Ulrike Dinger (Prof; MD), Alexander Bäuerle (PHD), Martin Teufel (Prof; MD) | Title page |
| 1. Occupation | **JBK, CJ, TL, LM:** research associate  **AB:** postdoctoral researcher  **EMS, WL, UD, MT:** full-time professors | p.3 |
| 1. Gender | Woman: JBK, TL, UD, EMS  Man: LM, WL, AB, MT, CJ | N/A |
| 1. Experience and training | **JBK:** educational background in psychology (M.Sc.), doctoral researcher  **CJ:** medical doctor, doctoral researcher  **TL:** educational background in psychology (B.Sc.), doctoral researcher  **LM:** educational background in psychology (B.Sc.), research assistant  **WL:** full-time professor in clinical psychology and psychotherapy  **UD:** full-time professor for psychosomatic medicine and psychotherapy  **AB:** postdoctoral researcher and head of research for psychosomatic medicine and psychotherapy  **MT:** full-time professor for psychosomatic medicine and psychotherapy  **EMS:** full-time professor for psychosomatic medicine and psychotherapy | p.3 |
| ***Relationship with participants*** | |  |
| 1. Relationship established | Participants consisted of two university medical centers for psychosomatic medicine and psychotherapy. No prior relationship established to the facilitator. | p.5 |
| 1. Participant knowledge of facilitator | The participants in the present study did not know the interviewer prior to the study. | p.5 |
| 1. Facilitator characteristics | No other characteristics were reported about the facilitator | p.5 |

| **Domain 2: Study design** | | **Reported on** |
| --- | --- | --- |
| ***Theoretical framework*** | |  |
| Methodological orientation and theory | Qualitative thematic analysis by Braun and Clarke (2006) | p.5 |
| ***Participant selection*** | |  |
| Sampling | Recruiting from 07-11-2023 to 01-19-2024. Purposive sampling. | p.4 |
| Method of approach | Recruiting via Email | p.4 |
| Sample size | *N* = 20 participants | *Table 1* |
| Non-participation | No responses from 3 individuals | p.3 |
| ***Setting*** | |  |
| Setting of data collection | Digitally and face-to-face | p.5 |
| Presence of non-participants | No presence of non-participants. | p.5 |
| Description of sample | Between 25 and 57 years (*M* = *35,05; SD* = *8,92*), 18 females, 2 males. See Table 1. | Table 1 |
| ***Data collection*** | |  |
| Interview guide | Provided as supplemental material | S1 |
| Repeat interviews | None | N/A |
| Audio/visual recording | Audio recording | p.5 |
| Field notes | Yes | p.5 |
| Duration | 18 - 36 minutes | p.5 |
| Data saturation | Yes | p.5 |
| Transcripts returned | No | p.5 |

| **Domain 3: Analysis and findings** |  | Reported on |
| --- | --- | --- |
| Number of data coders | Dual coding by TL and SB and cross-coding by JBK | p.5 |
| Description of coding tree | Yes (in the results section) | p.6 |
| Derivation of themes | (1) integration into psychotherapeutic work  (2) integration into clinical routines  (3) possible pitfalls of ROM implementation | p.6 |
| Software | MaxQDA 2024 | p.5 |
| Participant checking | No | N/A |
| ***Reporting*** | |  |
| Quotations presented | Yes | S3 |
| Data and findings consistent | Yes | S3 |
| Clarity of major themes | Yes | Fig.2 |
| Clarity of minor themes | Yes | Fig.2 |

**Reference:** Tong A, Sainsbury P, Craig J. Consolidated criteria for reporting qualitative research (COREQ): a 32-item checklist for interviews and focus groups. *International Journal for Quality in Health Care*. 2007. Volume 19, Number 6: pp. 349 – 357

**Table S3**

*Quotes for themes and subthemes*

| **Subtheme 1.1:**  **Guide for psychotherapy** | [1] “I think it's a good tool to get a first impression at the beginning and also to play along a bit with the course of therapy.” (11. Interviewee)  [2] “I think this is a good idea to give the course of therapy a bit of structure.” (12. Interviewee)  [3] “(ROMs) are important and perhaps bring a little more focus to the therapy. Keyword red thread.” (14. Interviewee)  [4] “So this is the chance and the greatest opportunity that we have with ROMs, that we can get away a little bit from the we assume that it works to we know that this and that works well and this or that works less well and it will also give us the opportunity to ask other questions. So that we no longer just have to ask what actually works, but that we can also ask a bit better, what works for whom, because we will suddenly have more options in heterogeneity and will no longer just see one for all but will also be able to find more personalized medicine.” (1. Interviewee)  [5] It's always a relief when, as I've just described, I perhaps didn't have the feeling that something was wrong with the patient's therapy. That I had the feeling that the therapy was perhaps progressing quite well and that there was a discrepancy with the patient's perception, then it is certainly very helpful. Especially for me as a beginner to see that (10. Interviewee).  [6] Yes, I believe that such continuous feedback can also reflect the relationship. As I said, you can use it as a conversation starter if you refer to it in individual therapy. […..] Um, but yes, I think it can provide a good structure for newcomers to the profession or in general, as well as an introduction to the conversation, and can be understood if it is well mapped (13. Interviewee). |
| --- | --- |
| **Subtheme 1.2:**  **Treatment monitoring** | [7] "You also look at the lab values relatively often or you monitor any other values like an ECG or whatever. And why shouldn't we do that on a psychometric, diagnostic level?" (5. Interviewee)  [8] “I think it's a good way to control the course of therapy or something like that.” (2. interviewee) |
| **Subtheme 1.3:**  **Change mechanisms** | [9] “The greatest added value is simply that we get an insight into something that was previously a bit of a black box.” (1. Interviewee)  [10] „It is decisive for the patients, not whether one therapy component worked well, but whether it is effective overall and if so, how well.” (15. Interviewee) |
| **Subtheme 1.4:**  **Self-reflection through**  **feedback** | [11] “Some patients may also find it more stressful. I don't know, but that's how we establish an introspection, where patients then think again, pause for thought. This is also something that has a therapeutic effect and where we have to make sure that it works in the right direction (8. Interviewee)”  [12] "I noticed when I started that I became a bit more vigilant when it came to dealing with patients." (2. Interviewee)  [13] "You often have a distorted perception of how the therapy process is going and sometimes the therapist has the impression that it's going much better than the patient would think." (2. Interviewee) |
| **Subtheme 1.5:**  **Patient & therapist**  **relationship** | [14] “At the moment I see it more as a feature that can perhaps be quite positive for my therapeutic development and also for the relationship with the patient” (10. Interviewee)  [15] “I am of the opinion that this actually strengthens the therapeutic relationship overall. That you reflect more on the therapeutic relationship and therefore also have a tool to reflect on this with patients” (7. Interviewee) |
| **Subtheme 2.1:**  **Quality assurance** | [16] "So to include this in the therapy process in order to perhaps also have objectifiability for the practitioners, but also for the patients." (3. Interviewee)  [17] "And we no longer have to rely solely on this therapeutic sovereignty, where we have a feeling and then make ourselves like this, but that we have measured this objectively. So I think objectivity really is the big opportunity." (1. Interviewee) |
| **Subtheme 2.2:**  **High Workload** | [18] “And I already know that there's a lot to do in everyday life on the ward and that's just on top of everything else, so to speak.” (16. Interviewee)  [19] “Of course, it's extra work for me because I have to look at the answers to the questionnaires. And then I also have to evaluate it for myself.” (14th interviewee)  [20] “But I think the structured integration of this into everyday working life is questionable. I'd say it's an extra effort at the beginning because it's not yet so clear. And I think we need to take a good look at that.” (5. interviewee)  [21] “But I believe that if you do the right frequency and don't choose these huge tests, it will bring many advantages and I would be prepared to put in more work for that." (6. Interviewee) |
| **Subtheme 2.3:**  **Supervision** | [22] "I would definitely need comprehensive training for employees, including myself, on how to interpret this." (5. Interviewee)  [23] "And that's why I would plan for these questionnaires to be firmly integrated into the inpatient stay, so that patients can see them and ask questions, and the results can be discussed." (10. Interviewee)  [24] "And that means, as I have already said in part, that it would be somehow important for them to be integrated into supervision." (17. Interviewee) |
| **Subtheme 2.4:**  **Structural organization** | [25] „It would ultimately be a new tool that would have to be implemented and integrated into the processes.” (14. Interviewee)  [26] "I think it needs the structure where it routinely belongs. It needs structuring. Certain periods of time during the day, so that it's marked in the daily routine, plan, patients' timetable, what exactly time is set aside for it, because otherwise it goes down quickly." (8. Interviewee)  [27] “However, I think it would be good for the patients if they had a quiet room like this, where they could do other tasks, but also fill in the ROMs and there's no talking in between or anything else. So that they can find a place to do it in peace.” (6. interviewee)  [28] “That's a bit of a problem. We don't have that. We have therapy-free times, but no rooms. That's a difference.” (5. interviewee) |
| **Subtheme 2.5:**  **Transparent discussion with**  **patients** | [29] “I think it's always very important to discuss this with the patient, because they've completed the questionnaire and naturally want to know how they're getting on, so to speak, and discussing this often has a therapeutic purpose.” (6. Interviewee)  [30] "I have experienced that when tests are conducted without subsequent discussion or reflection, it creates significant uncertainty among patients. They start to question, 'What are they doing with this? Why did they test me? Are they testing themselves? Are they testing the situation?' There are so many questions that arise from this, which is why I always strive to disclose the results with the utmost transparency" (7. Interviewee). |
| **Subtheme 3.1:**  **Perceived performance**  **pressure** | [31] “Well, for example, that therapists actually feel controlled, that patients feel controlled, what you do with these results. I mean, we have now, we are now here at the LVR, but there are also, I'll put it this way, private, commercially oriented providers, you could ask yourself whether there are consequences in terms of personnel planning and so on. So it's like a performance evaluation. Maybe you can say misinterpretation as a performance evaluation of the employees” (15. Interviewee)  [32] “I don't believe in surveillance at all. If this feeling arises, you just have to talk about it properly so that it doesn't happen. You have to deal with it” (8. Interviewee).  [33] “I can understand the concern, of course. Nobody wants to be a therapist on a ward where it somehow always comes out at the end that the therapy supposedly didn't help.” (15. Interviewee)  [34] Yes, that's exactly what I mean. I mean, what would be the logical step. We do speak about patients in the team. Not always all of them. Mostly specific points and that there are none. You can then add that it's also part of the process to talk about the relationship. The question is then whether every therapist feels comfortable to enter the evaluation of themselves in the team (13. Interviewee) |
| **Subtheme 3.2:**  **Restriction of therapy to**  **ROM-data** | [35] “There is a concern that your own success will be measured by this, then of course you may start to only work with the patient when depression is queried, even though they might actually have two or three issues that would be more urgent, but are more individual and are not queried.” (15. Interviewee)  [36] “And then you just have to see how to incorporate it cleverly and whether you can then basically design the whole lesson based on it.” (14. Interviewee) |
| **Subtheme 3.3:**  **Inconclusive data**  **interpretation** | [37] “Have progressions displayed, which are then much easier to discuss with the patient.” (2. interviewee)  [38] “I'm actually worried that this will somehow lead to competition, but there may already be some comparability.” (4. interviewee) |
| **Subtheme 3.4:**  **Risk for patients** | [39] “My worry would be that people would try to present themselves better afterwards and do better.” (4. Interviewee)  [40] And of course you can't prevent someone from using that to say that they think everything here is terrible and then ticking all the boxes to say that nothing has helped (15. Interviewee).  [41] Well, that can of course be an obstacle for patients in many different situations. Just as patients experience various things in therapy as inhibiting therapy because a psychopathology is produced, for example, so that patients tend to see themselves in the difficulty of not being able to enter into relationships well because there is a third party in the relationship, so to speak, namely ROM (1. Interviewee)  [42] “If patients have such structural difficulties that simply performing regular ROMs is too much of a challenge.” (1. Interviewee)  [43] “As I understand it, it also reflects the doctor-patient therapeutic relationship. And there are certainly difficult relationships where, simply because of the disorder, the patient perhaps doesn't feel seen enough or perhaps feels too limited, which we also notice here on the ward, but where there is actually also a small concern. Of course, that will be reflected.” (4. Interviewee)  [44] "One thing is that, especially with patients who have difficulty concentrating, it's simply exhausting for them to sit down and fill it out, because they might also be dealing with exhaustion." (3. Interviewee)  [45] „Our patients also repeatedly report that filling out the questionnaires is very strenuous.“ (20. Interviewee) |
